# The effect of e-mental health interventions on academic performance in university and college students: A meta-analysis of randomized controlled trials

**DOI:** 10.1016/j.invent.2020.100321

**Published:** 2020-04-23

**Authors:** F. Bolinski, N. Boumparis, A. Kleiboer, P. Cuijpers, D.D. Ebert, H. Riper

**Affiliations:** a Department of Clinical, Neuro-, and Developmental Psychology, Vrije Universiteit Amsterdam, the Netherlands; b Amsterdam Public Health Research Institute, Faculty of Behavioral and Movement Sciences, Vrije Universiteit Amsterdam, the Netherlands; c Department of Clinical Psychology and Psychotherapy, Friedrich-Alexander-University Erlangen-Nuremberg, Germany

**Keywords:** Students, College, University, E-mental health, Academic performance, Meta-analysis

## Abstract

**Background:**

Mental health symptoms are common among college and university students and these can affect their academic performance. E-mental health interventions have proven effective in addressing mental health complaints but their effect on academic performance has not been synthesized yet.

**Objectives:**

To synthesize the evidence from randomized controlled trials for the effectiveness of e-mental health interventions on academic performance in college and university students compared to inactive controls.

**Data sources and eligibility criteria:**

We searched six databases (PubMed, Cochrane library, CINAHL, ERIC, PsycINFO, Web of Science) during the period January 2000 until September 2019 for randomized controlled trials that reported on e-mental health interventions (guided or unguided) for college and university students and measured academic performance (e.g. grade point average).

**Study appraisal and synthesis methods:**

Study and participant characteristics and the academic performance measures at post-intervention were extracted. The latter were pooled and Hedges' *g* was calculated as the effect size. Heterogeneity and publication bias were investigated.

**Results:**

Six studies containing 2428 participants were included in the meta-analysis. These focussed on either mood and anxiety or alcohol and tobacco use. The pooling of data resulted in a small but non-significant effect of *g* = 0.26 (95% CI, −0.00, 0.52; *p* = .05) on academic performance, favouring e-mental health interventions over inactive controls. Interventions had positive effects on depression (*g* = −0.24) and anxiety (*g* = −0.2). Heterogeneity was high.

**Discussion:**

Despite the small and non-significant effect, our meta-analysis points to a promising direction for the effectiveness of e-mental health interventions on academic performance. Yet, these results must be interpreted with caution, as heterogeneity was high and few studies on the effectiveness of e-mental health interventions for students reported academic performance measures.

## Highlights

• *E*-mental health interventions had a small but statistically non-significant effect on academic performance (*g*= .26; *p*= .05).
• They had small and statistically significant effects on depression (*g*= -.24; *p*= .03) and anxiety (*g*= -.2; *p*< .01 ).
• The majority of screened studies did not report on measures of academic performance.

## 1 Introduction

The college and university years constitute a crucial period in young adults' lives. Increased personal freedom often goes hand in hand with a variety of stressors, such as a change in social support structures, financial independence, and new living arrangements (Thurber and Walton, 2012). According to large scale epidemiological studies, annually around 20% -30% of college and university students (henceforth denoted as students) suffer from any common mental health condition, such as mood and anxiety disorders (Auerbach et al., 2016; Ibrahim et al., 2013).

The presence of mental health conditions has often been linked to impaired academic achievement in college and university. For example, Hysenbegasi et al. (2005) collected annual data from their university registrar's office and showed that a diagnosis of depression was significantly associated with the loss of half an overall performance grade within one semester. Given that grade point average (GPA) thresholds are often represented in 0.5 point intervals, this association is noteworthy. Moreover, though largely based on cross-sectional studies, associations between suicidal ideation, depression and lower grades have been found continuously among students (Andrews and Wilding, 2004; De Luca et al., 2016). Mortier et al. (2015) showed that at the end of the first academic year, the final grade percentage (i.e. the weighted sum of all grades) of students who attempted suicide before entering university was around 8% lower compared to those freshmen students who did not attempt suicide. Finally, a longitudinal study by Eisenberg et al. (2009) suggests that depressive and anxiety symptoms are not only related to lower grades, but also to an increased probability of discontinuing college. In the context of substance use, Arria et al. (2013) showed that over a four year period, around 40% of frequent marijuhana users dropped out of college, compared to around 25% of minimal users.

Tackling mental health issues in students can thus be beneficial for the individual student and society at large, not least because in turn academic output can be improved. That is, attainment in college is one of the driving factors for the accumulation of human capital (Brand and Xie, 2010; Hasan and Bagde, 2013). This is defined as factors, such as knowledge, that are needed in order to be productive in the labour market (Goldin, 2016). Moreover, higher grades in college have been associated with larger financial earnings in the work setting and lower risk of unemployment (Kittelsen Røberg and Helland, 2017). Conversely, improving students' mental health could positively affect their economic outlook, potentially through improving their academic performance.

Mental health interventions provided on a computer or via the Internet, which we will henceforth denote as e-mental health interventions, have acquired a solid empirical basis for the prevention and treatment of various psychological conditions in adults, including depression (Buntrock et al., 2016; Karyotaki et al., 2017), anxiety (Grist et al., 2019), and alcohol use (Riper et al., 2018). In addition to these, a significant number of randomized controlled trials (RCTs) on e-mental health interventions have been carried out in student populations. In their systematic review and meta-analysis, Davies et al. (2014) synthesized the effects of e-mental health interventions, the majority being browser-based and including some form of human guidance, for improving students' mental health. They identified 17 RCTs investigating 14 interventions, the majority of which based on cognitive behavioural therapy (CBT), with a total of 1480 participants. With the exception of one, which aimed at treating social phobia (Botella et al., 2010), the included studies focused on the prevention of depression, anxiety (general, social, examination), and stress. The authors found that compared to inactive control conditions, e-mental-health interventions had a moderate effect on symptoms of depression (*d* = 0.43; nine studies; 712 participants), anxiety (*d* = 0.56; seven studies; 374 participants), and stress (*d* = 0.73; three studies; 217 participants). However, the pooling of data of the two comparisons (229 participants) with active control conditions, such as online psychoeducation material, did not result in a significant difference between e-mental health interventions and controls. In a more recent meta-analysis of 48 RCTs on this topic, Harrer et al. (2018a) investigated the effects of e-mental health interventions for common mental health conditions and stress, as well as on wellbeing in students compared to inactive controls. With the exception of wellbeing, their results showed significant small to moderate effects favouring the e-mental health interventions, specifically for depression (*g* = 0.18), anxiety (*g* = 0.27), disordered eating (*g* = 0.52), and stress (*g* = 0.2).

As of yet, research on whether these effects extend to academic performance is largely limited to interventions delivered offline. For example, Conley et al. (2015) pooled academic performance data from 90 studies with 103 individual face-to-face universal mental health prevention interventions. This resulted in a statistically significant but small effect size of *g* = 0.18 (*p* < .01). In their randomized controlled pilot study, Melnyk et al. (2015) tested whether an Internet-based cognitive behavioural therapy (ICBT) intervention was effective in tackling students' symptoms of depression, anxiety, and whether its use positively affected their grade performance measured at the end of the academic year. They found that students with severe anxiety at baseline who were randomized into the intervention group reported significantly fewer symptoms of depression and anxiety, whereas those in the control group did not. Moreover, students who received the intervention had statistically significant higher grade-point average (GPA) scores at the end of the semester compared to those in the control group (3.58 vs. 3.28 respectively; *p* = .02).

Based on the suggested association between mental health and academic performance, the aim of the current study was to assess the pooled effectiveness of e-mental health interventions compared to inactive controls for improving academic performance in students. We also aimed to pool the effects on mental health outcomes of the included studies.

## 2 Methods

### 2.1 Identification of studies

A review by Andersson (2016) located the onset of RCTs on web-based interventions for mental health complaints around the year 2000. Our bibliographical searches were thus limited to records published between January 2000 and September 2019. The subsequent screenings were completed independently by F.B. and N.B. Any disagreement was solved by discussion, where necessary with senior researchers (A.K., H.R.). Initially, we screened the titles and abstracts and retained studies that potentially met our inclusion criteria. The references of all included studies were checked for additional relevant records. All searches were performed in six databases. These were selected because they covered the fields of education (ERIC), as well as clinical trials and intervention studies (CINAHL, Cochrane Library, PsycINFO, PubMed, Web of Science). The search strings were compiled of terms for academic performance (e.g. marks, GPA), students (e.g. college, university), e-mental health interventions (e.g. online, e-health, Internet-based), and filtered to include only RCTs. Index terms were used if available and these were complemented by free-text terms. The complete search string for PubMed is provided as Supplementary material. Since we expected that academic performance was not always used as primary outcome and therefore not reported in title or abstract, we screened reviews on e-mental health interventions for students (Harrer et al., 2018a) for additional studies.

### 2.2 Eligibility criteria and data extraction

**Fig. 1:**
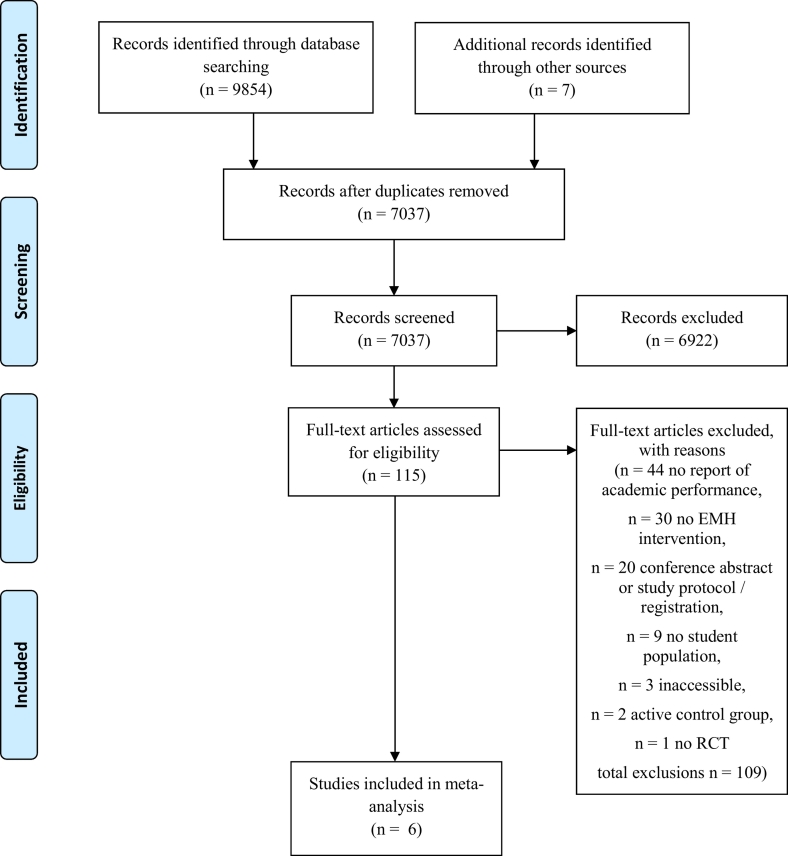
Flowchart.

Studies were deemed eligible if they reported on students (graduate or undergraduate), that were enrolled at their institution at the time of the outcome assessment. These institutions could refer to any form of higher education, such as colleges, universities, or universities of applied sciences. Moreover, participants had to be randomized either into a group receiving an e-mental health intervention, which was defined as any intervention that a) was either Internet-based or computerized, and b) had a focus on the improvement of mental health (e.g. depression, anxiety, substance misuse) or into an inactive control group (e.g. waitlist, assessment only). Furthermore, academic performance had to be reported on after the intervention was administered, regardless of the type of variable (e.g. outcome, moderator, mediator) and whether this was self-reported or retrieved from the institutes' administration. Extracted variables included a) test scores of individual courses (e.g. mid-term), b) final grades of individual courses, or c) average semester grades (i.e. GPA). Based on the nature of these academic performance variables, no distinction could be made between immediate and delayed assessment and therefore it was not possible to accurately determine the time that elapsed between the end of the intervention and the academic performance assessment. For example, exam grades were either collected in the middle or at the end of a course, whereas GPA is usually calculated at the end of a semester. Where sufficient information on these outcome variables could not be extracted from the published record, we contacted the first author in order to retrieve the missing information. If the first author could not be reached or did not reply, we contacted the last author. The number of inaccessible studies, meaning where no contact could be established or where data was unavailable, is presented in Fig. 1.

Next to post-intervention assessments of academic performance, we also extracted other characteristics of the individual studies (see Table 1). These referred to in- and exclusion criteria, country, the type of intervention and control condition (i.e. waitlist vs. assessment only), their sample sizes, and the mental health outcomes. Extraction of data from the published records was done independently by two researchers (F.B., N.B.). Any disagreement was solved by discussion. We summarized the number of all included studies and the total number of participants in the intervention and control groups. Next, we created clusters based on the primary focus of the intervention and summarized core information per cluster. A description of the intervention used in each study can be found in Table 2.

**Table 1:** Characteristics of the individual studies included in the meta-analysis.

Study	Participants	Mean age (SD)	Intervention	Comparator	AP measure	MH measure	Country	Inclusion criteria	Exclusion criteria	N_int_	N_con_
Epton et al. (2014)	Freshmen students	18.9	ATI	AO	Exam	AUDIT	UK	IC	None reported	473	481
Gilbertson et al. (2018)	Freshmen students	18.02 (0.29)	ATI	WL	GPA	QFI	USA	≥ 18 years	None reported	32	27
Melnyk et al. (2015)^a^	Freshmen students	18.7 (4.1)	MAI	AO	GPA	PHQ-9; GAD-7	USA	≥ 18 years	None reported	42	22
Shin (2013)^a^	Freshmen students	18.3	MAI	AO	GPA	DASS	USA	None reported	None reported	62	76
Viskovich and Pakenham, (2019)	All students	26.85 (8.77)	MAI	WL	GPA	DASS	AUS	≥ 18 years; course enrolment; fluent English	None reported	596	566
Wolitzky-Taylor and Telch, (2010)	Clinically worried students	Not reported	MAI	WL	GPA	PSWQ	USA	Worry causes significant interference with life or distress (based on AWQ, not further specified)	Taking psychotropic medication/not staying on same dose/planning to start/terminate psychotherapy; epilepsy/seizures	33	18

Note. ATI = Alcohol and tobacco smoking intervention, MAI = mood and anxiety intervention, AO = assessment only, WL = waitlist, GPA = grade point average, AUDIT = Alcohol Use Disorder Identification Test (Saunders et al., 1993), QFI = Quantity and Frequency Index (Gilbertson et al., 2018; Haberman, 1970), PHQ-9 = Patient Health Questionnaire (Kroenke et al., 2001), GAD-7 = Generalized Anxiety Disorder questionnaire (Spitzer et al., 2006), DASS = Depression and Anxiety Stress Scale (Lovibond and Lovibond, 1995), PSWQ = Penn State Worry Questionnaire (Meyer et al., 1990), IC = Informed Consent, AWQ = Academic Worry Questionnaire (Wolitzky and Telch, 2005) N int = number of participants in the intervention group, N con = number of participants in the control group.

a Data received by author.

**Table 2:** Description of interventions of the studies included in the meta-analysis.

Study	Intervention	Sessions	Duration (minutes/session)
Epton et al. (2014)	U@Uni: composed of a web-based platform and could additionally be used on an associated app. Before accessing the intervention, students had to identify their most important value (e.g. humour, respect) and the reason why this value is so important to them. This was based on self-affirmation (Epton et al., 2013), which is used to reduce defensive reactions to the content of the intervention. The platform used videos and informative texts, as well as an online planner to promote the targeted health behaviours. Moreover, standardized motivational messages were used to encourage adoption of healthy behaviours (i.e. discouraging drinking).	nr	nr
Gilbertson et al. (2018)	Alcohol-wise: based on personalized normative feedback (PNF) and students in the intervention group received log-in instructions via email from the central university administration.	nr	nr
Melnyk et al. (2015)	Creating Opportunities for Personal Empowerment (COPE). An adaptation of a face-to-face CBT protocol, which was integrated into the university's online course platform. Content included videos and exercises. Module 1: introduction; Module 2: Self-esteem; Module 3: Healthy coping and reducing stress; Module 4: Goal setting and problem solving; Module 5: Emotions, positive thinking, and communication; Module 6 and 7: Revision and summary.	7	30
Shin (2013)	Self-developed intervention aimed at enhancing students' meaning in life with an assumed positive effect on depression. It contained four consecutive modules: Module 1: Introduction and goal setting; Module 2:Personal strengths and career plan for meaningful work; Module 3: I Integration of knowledge about oneself with meaningful goals..; Module 4: Wrap-up session	4	30
Viskovich and Pakenham, (2019)	You Only Live Once intervention (YOLO): based on Acceptance and Commitment Therapy (ACT), which covers the main aspects of this treatment approach: module 1: clarifying values and goals according to the SMART acronym; module 2: cognitive defusion; module 3: acceptance; and module 4: mindfulness.	4	30–45
Wolitzky-Taylor and Telch, (2010)	Computerized expressive writing intervention: based on the original treatment paradigm by Pennebaker and Beall (1986). The computerized writing sessions were preceded by an in-person instruction session. Within the writing sessions, students were encouraged to give a detailed account of their academic fears.	nr	nr

Note. nr = not reported.

### 2.3 Power calculation

We conducted a power calculation on the number of studies needed in order to obtain enough statistical power for detecting post-test effect sizes. We assumed the power to be *β* = 0.8, a significance level *α* = 0.05, and a moderate degree of between-study variance *τ*^2^ as described by Borenstein et al. (2009). Since effect sizes for academic performance in e-mental health interventions have not been established in the context of higher education, we used the standardized mean difference of *g* = 0.18 reported in the meta-analysis by Conley et al. (2015) as reference. We would therefore need five studies with around 160 participants per condition, or ten studies with around 80 participants per condition (Borenstein et al., 2009). This translates to five studies with around 22 participants per group or ten studies with around 11 participants per group in order to detect a moderate effect size of *g* = 0.5 (Borenstein et al., 2009; Cohen, 1988).

### 2.4 Quality assessment

We used the revised Cochrane risk of bias assessment tool (Higgins et al., 2011) to evaluate the methodological quality of the included studies. The following criteria were rated as either low risk of bias, high risk of bias, or unclear risk of bias: a) adequate generation of random sequence, b) proper allocation concealment, c) blinding of participants and personnel, d) blinding of outcome assessment, and d) adequately addressing attrition bias by intention-to-treat (ITT) analysis or multiple imputation. The risk of bias assessment was completed independently by two researchers (F.B., N.B.) with any disagreement solved by discussion.

### 2.5 Meta-analysis

Meta-analyses were conducted on academic performance and mental health outcomes. If not otherwise specified, all analyses were run in Comprehensive Meta-Analysis version 3 (CMA; Borenstein et al., 2013). We calculated Hedges' *g* as the difference in means between the intervention and control condition for each comparison, divided by the pooled standard deviation and adjusted for small sample bias (Hedges and Olkin, 1985) by transforming means, sample sizes, and *p*-values, or if this information was not available, by transforming reported effect sizes (e.g. Cohen's *d*; Cohen, 1988).

A considerable degree of heterogeneity between the studies was expected. We therefore conducted the analyses under a random-effects model, which assumes that the included studies differ significantly from each other. First, heterogeneity was visually inspected by using the forest plot and statistically through the *I*^2^ statistic, which expresses the degree of heterogeneity from 0% to 100%. An *I*^2^ of around 25%, 50%, and 75% can be interpreted as low, moderate, and high heterogeneity respectively (Higgins et al., 2003). 95% confidence intervals (CI) around *I*^2^ were calculated in Stata using the HETEROGI module (Orsini et al., 2006). Moreover, where outlying studies were present, we conducted a sensitivity analysis by excluding those studies to investigate the extent to which they influenced the results. Outliers were identified by comparing the overlap of the 95% CI of the individual studies' effect size with the 95% CI of the pooled effect size. Publication bias was investigated by visually assessing the symmetry of the funnel plot and by conducting Egger's test of the intercept with a one-tailed significance level *α* = 0.05. The latter is a regression-based approach that tests if statistically significant bias exists in favour of the intervention by predicting the standardized effect size of the included studies from the inverse of their standard error (i.e. their precision; Egger et al., 1997). Moreover, we used Duval and Tweedie's trim and fill procedure (Duval and Tweedie, 2000). This method removes small studies that potentially cause asymmetry in the funnel plot. Based on this trimmed plot, these studies and those that are missing on the other side of the re-estimated centre are subsequently imputed.

Lastly, we ran two univariate meta-regression analyses. First, in order to assess whether the clinical effectiveness of the e-mental health interventions, that is their effect on the mental health outcomes, could predict their effect on academic performance. The extracted effect sizes on mental health outcomes served as the predictor. The derived regression coefficient indicates the degree of change in the pooled effect size on academic performance with a one-unit increase in clinical effectiveness. This can be interpreted as the in- or decrease in academic performance with one additional standard deviation difference in clinical effectiveness between the intervention and control group. Second, in order to investigate whether the effect size of e-mental health interventions could be predicted by the degree of risk of bias of the individual studies. For this meta-regression analysis, we chose a conservative approach by combining ratings of unclear and high risk of bias. The regression coefficient indicates the extent to which the pooled effect size changes with a one-unit increase in methodological quality, that is with one additional criterion rated as low risk of bias.

## 3 Results

### 3.1 Selection and inclusion of studies

The bibliographic searches resulted in a total of 9854 records and searches of references yielded an additional seven records. Of these, 7037 remained after the deletion of duplicates. Another 6922 records were excluded after scanning their titles and abstracts. The full texts of the remaining 115 records were retrieved and assessed for inclusion, among those eight for which authors had to be contacted in order to acquire data or the full record. Six studies with six individual comparisons and 2428 participants (n = 1238 in the experimental and n = 1190 in the control conditions) met all inclusion criteria. Fig. 1 depicts the flowchart of the inclusion process.

### 3.2 Characteristics of the included studies (*N* = 6)

An overview of the characteristics of the six studies can be found in Table 1. In one RCT, academic performance was reported as a primary outcome (Viskovich and Pakenham, 2019). For the other studies this could not be determined. Information on the participants' age was reported in all but one (Wolitzky-Taylor and Telch, 2010), ranging from 17 to around 27 years. All interventions were self-guided, meaning none provided human guidance beyond technical instructions and support. Concerning the academic performance measure, five reported GPA (Gilbertson et al., 2018; Melnyk et al., 2015; Shin, 2013; Viskovich and Pakenham, 2019; Wolitzky-Taylor and Telch, 2010) and one reported not further defined average exam scores (Epton et al., 2014). With the exception of two studies (Viskovich and Pakenham, 2019; Wolitzky-Taylor and Telch, 2010), the majority targeted incoming or freshmen students. Apart from Wolitzky-Taylor and Telch (2010), which required clinically significant worry for participation, no clear clinical in- or exclusion criteria were reported.

Four RCTs targeted mood and anxiety with 733 participants in the intervention and 682 participants in the control groups. Three of these used evidence-based protocols, namely cognitive behavioural therapy (CBT; Melnyk et al., 2015), acceptance and commitment therapy (ACT; Viskovich and Pakenham, 2019), and expressive writing (Wolitzky-Taylor and Telch, 2010). The remaining study focused on increasing meaning in life with the aim to reduce mental health complaints (Shin, 2013).

Two of the six studies specifically tackled the reduction or prevention of substance misuse (specifically alcohol and tobacco smoking) with 505 participants in the intervention and 508 in the control conditions. Both of these used a universal prevention approach for alcohol (Gilbertson et al., 2018) and alcohol and cigarette consumption (Epton et al., 2014).

### 3.3 Quality of studies

**Fig. 2:**
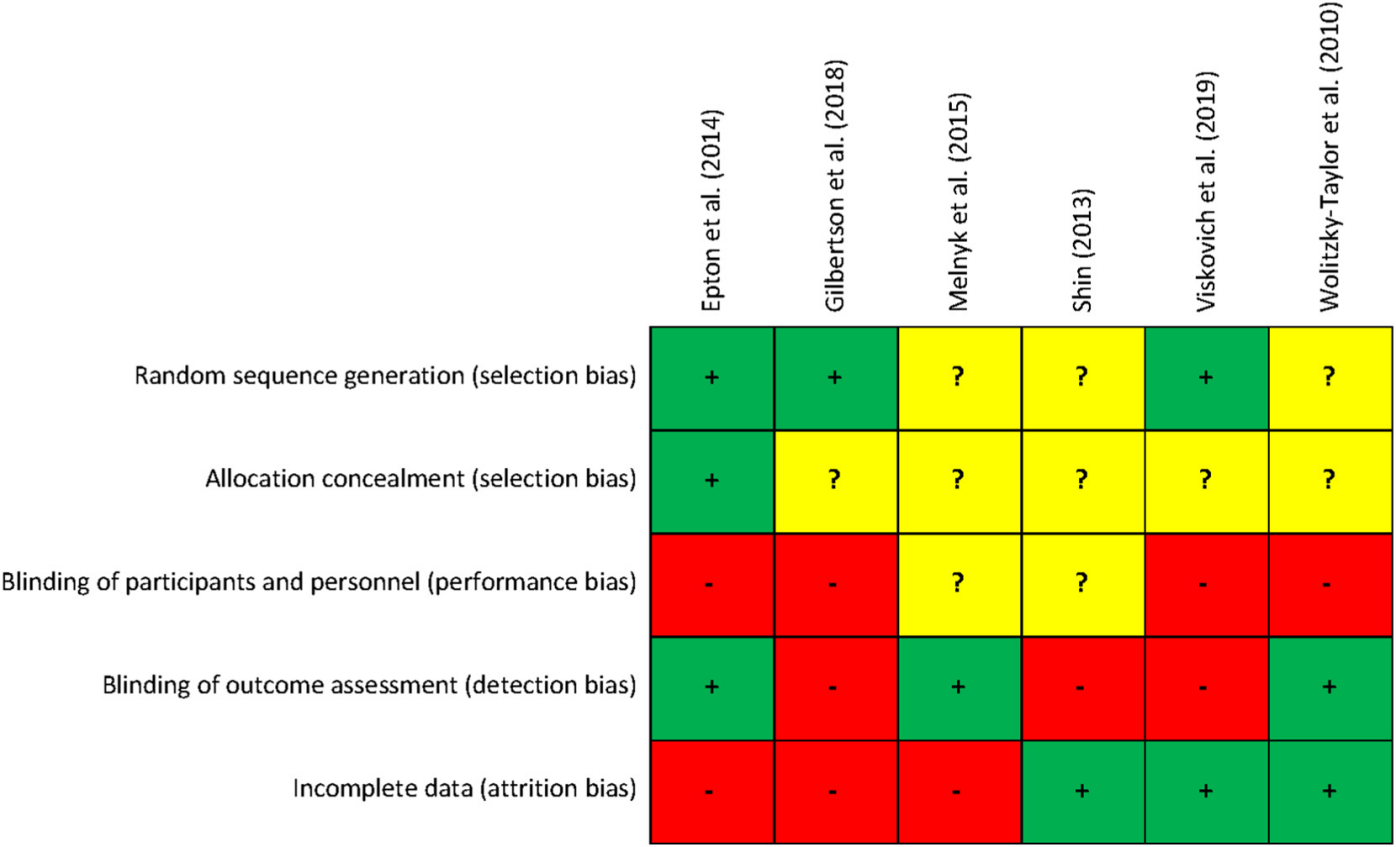
Individual risk of bias assessment of studies included in the meta-analysis.

The methodological quality of the six studies included in the meta-analysis is visually depicted in Fig. 2. Out of these, three clearly reported a random sequence generation. For the remaining three studies this was unclear. Allocation concealment could be determined for one RCT, with the remaining five again being rated as unclear. The blinding of participants and personnel was not done or not properly described in any of the studies; it was rated as high risk in four and unclear in two RCTs. Blinding of outcome assessment was considered adequate in three studies, as GPA and test scores were gathered from the central study administration. Another three were rated as high risk because self-reported GPA was used. Attrition bias was addressed appropriately in three of the six studies by reporting ITT analyses, whereas the remaining three used completers only data or last observation carried forward (LOCF) imputation. None of the studies fulfilled all five criteria. One did so for three, two for two, and three studies met one criterion.

### 3.4 Pooled post-intervention effects on academic performance

**Fig. 3:**
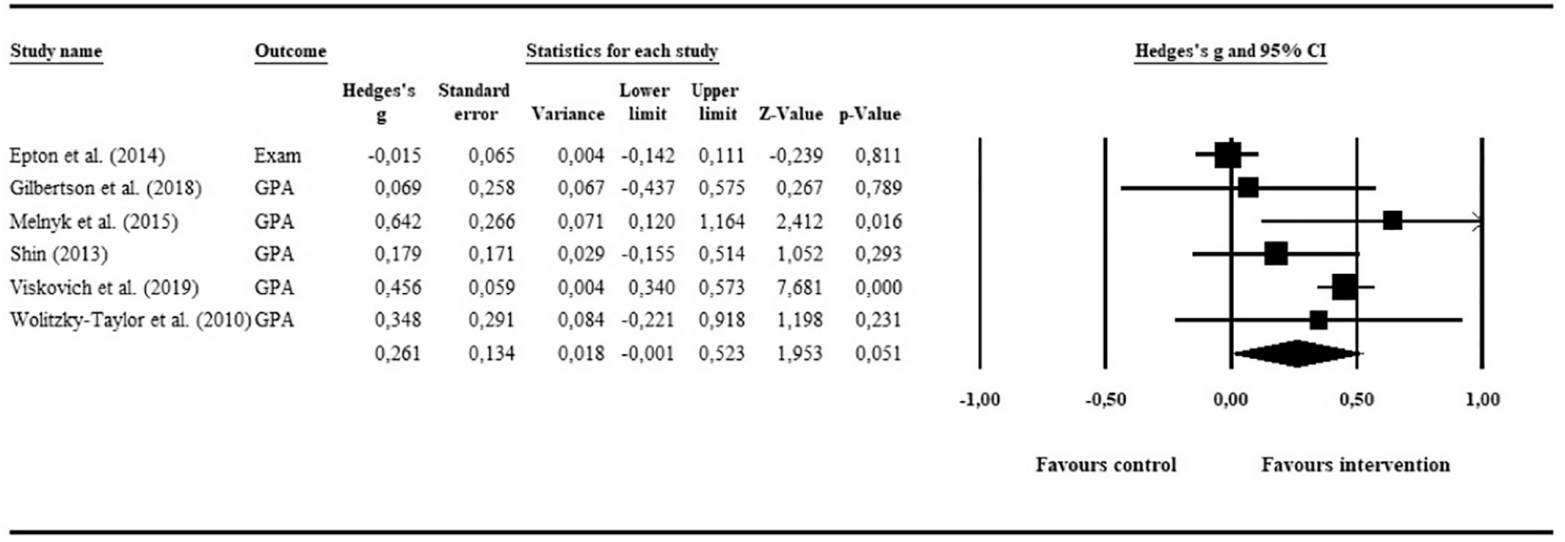
Meta-analysis of e-mental health interventions on academic performance.

The pooling of academic performance data yielded a small and non-significant effect of *g* = 0.26 (95% CI: −0.00, 0.52; *p* = .05) for e-mental health interventions compared to inactive controls. Fig. 3 shows the associated forest plot. No potential outliers were identified. The investigation of heterogeneity suggested considerable and statistically significant differences between the studies, with *I*^2^ = 84.3 (95% CI: 63, 91; *p* ≤ .001). Due to the insufficient number of comparisons, we were unable to conduct subgroup analyses in order to investigate reasons for heterogeneity.

The visual inspection of the funnel plot suggested potential publication bias, and one study was imputed using Duval and Tweedie's trim-and-fill procedure. The resulting imputed effect size dropped marginally (*g* = 0.21, 95% CI: −0.04, 0.46). However, Egger's test of the intercept was not significant (*p* = .46), suggesting little evidence for publication bias. Finally, only one of the six studies provided follow-up academic performance data that was suitable for pooling (Gilbertson et al., 2018). Therefore, the calculation of long-term effects was not possible.

### 3.5 Pooled post-intervention effects on mental health outcomes and meta-regression

#### 3.5.1 Depression

**Fig. 4:**
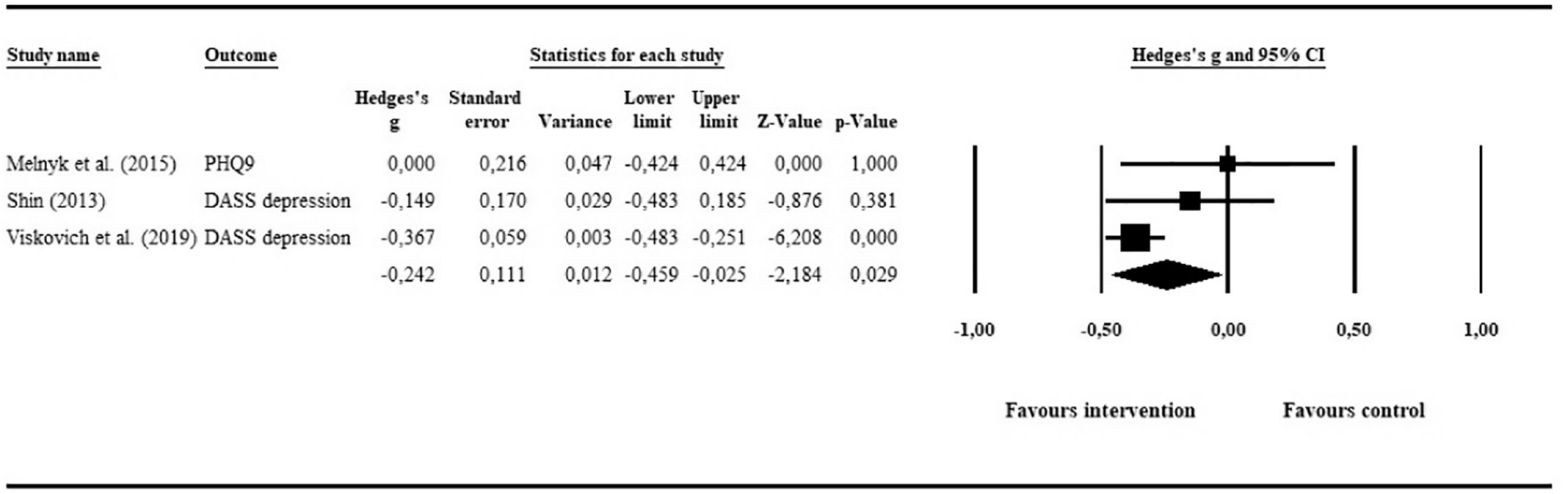
Meta-analysis of e-mental health interventions on depression outcomes.

Three RCTs (*N* = 719 in intervention group; *N* = 674 in control group) provided post-intervention data on depression. Pooling of data resulted in a statistically significant but small effect of *g* = −0.24 (95% CI: −0.46, −0.03; *p* = .03), favouring e-mental health interventions over inactive controls. The visual inspection of the forest plot (Fig. 4) did not suggest any outliers, as the 95% CIs of all individual studies' effect sizes overlapped with the 95% CI of the pooled overall effect size. Heterogeneity was moderate and statistically not significant, with *I*^2^ = 46.94 (95% CI: 0, 84; *p* = .15).

#### 3.5.2 Anxiety

**Fig. 5:**
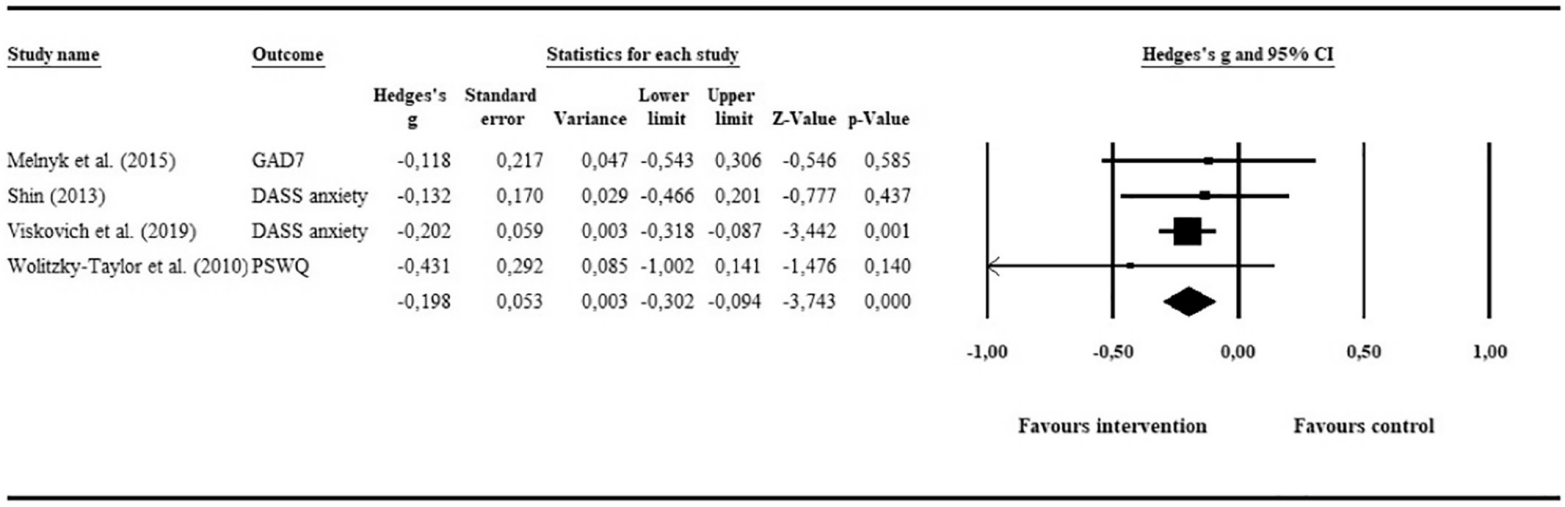
Meta-analysis of e-mental health interventions on anxiety outcomes.

Four RCTs (*N* = 752 in intervention group; *N* = 692 in control group) presented anxiety outcomes, the pooling of which yielded a statistically significant but small effect of *g* = −0.2 (95% CI: −0.3, −0.09; *p* ≤ .01) in favour of the intervention groups. Based on the forest plot (Fig. 5), no outliers were detected and heterogeneity was low and statistically not significant, with *I*^2^ = 0 (95% CI: 0, 68; *p* = .82).

#### 3.5.3 Alcohol consumption and tobacco smoking

**Fig. 6:**
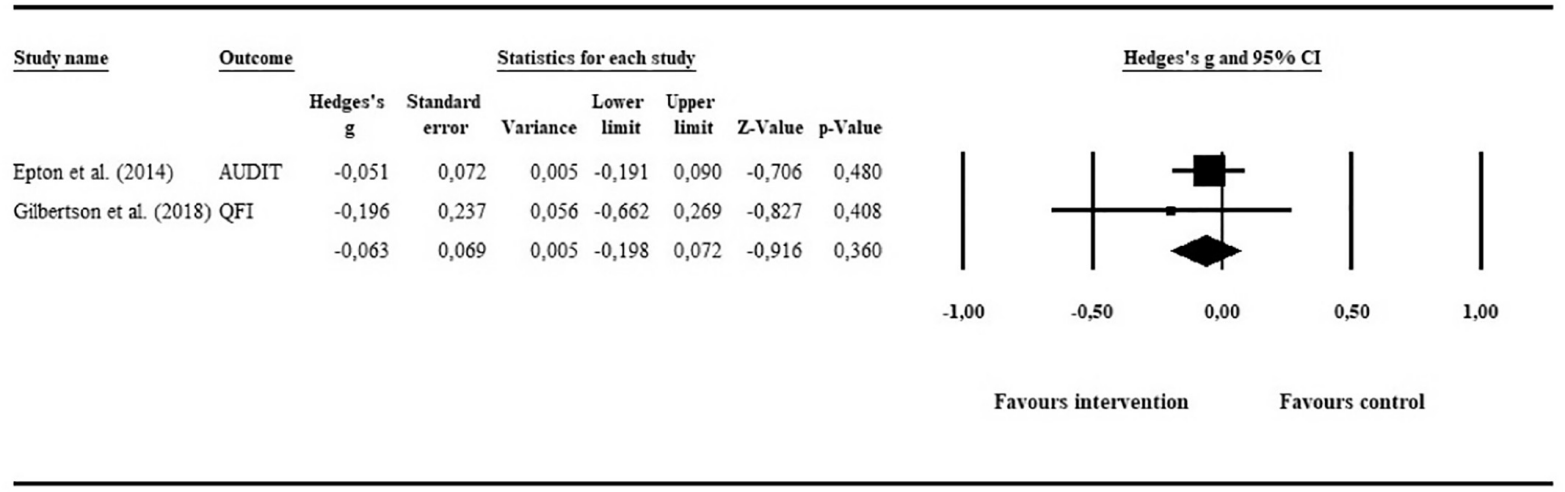
Meta-analysis of e-mental health interventions on alcohol consumption.

Two studies aimed at reducing alcohol consumption (*N* = 413 in intervention group; *N* = 433 in control group), one of these also reported post-intervention data on tobacco smoking (Epton et al., 2014; could not be pooled). The overall effect on alcohol-related outcomes was small and non-significant: *g* = −0.06 (95% CI: −0.20, 0.07; *p* = .36). Heterogeneity could not be investigated statistically (degrees of freedom <2). However, as shown in the forest plot (Fig. 6), none of the studies were considered outliers.

The effect size of mental health outcomes were not a significant predictor for academic performance (*b* = −0.95, 95% CI: −2.48, 0.58; *p* = .22). The same was the case for the meta-regression analysis using risk of bias as predictor (*b* = −0.12, 95% CI: −0.42, 0.18; *p* = .43).

## 4 Discussion

A positive relationship between students' mental health and their academic performance is assumed (Hysenbegasi et al., 2005; Mortier et al., 2015). Our primary aim was thus to assess the overall effect of e-mental health interventions on measures of academic performance in students by means of a meta-analysis. Since the underlying assumption is that these interventions also prove clinically effective, we also pooled data on mental health outcomes of the included studies. Six RCTs with 2428 participants were identified.

### 4.1 Main outcomes

The pooling of studies yielded a small and non-significant effect of *g* = 0.26 (95% CI: −0.00, 0.52; *p* = .05) on measures of academic performance, favouring the e-mental health interventions. An effect which, according to general conventions, is considered small (Cohen, 1988). However, these conventions have been generally applied to clinical outcomes and might thus poorly reflect improvements in academic performance. Evidence from elementary and high schools suggests that educational interventions (i.e. those aimed at improving academic performance) are expected to have much smaller, nonetheless meaningful effects, with effect sizes above *d* = 0.20 considered large (Kraft, 2019). Moreover, since none of the included studies provided human guidance other than technical support, their small effect may still be relevant from a public health perspective. On a population level, the gains could then be considerable, given that such unguided interventions can be scaled up at relatively low financial expense as there are no costs for therapists or coaches involved (Donker et al., 2009; Riper et al., 2014). In addition, students' vast access to and use of the internet (World Bank, 2020) form a strong basis for scaling up such unguided interventions, for example within the university infrastructure.

We also tested the basic assumption regarding the effectiveness of the included e-mental health interventions on mental health outcomes compared to inactive control conditions. Three of the six RCTs provided post-intervention outcomes on depression. Their pooled effect was *g* = −0.24 (95% CI: −0.46, −0.03; *p* = .03), favouring e-mental health interventions over inactive controls. Though small, this effect precisely encompassed the cut-off for clinical relevance as established by Cuijpers et al. (2014). The pooled effect of the four studies reporting on anxiety outcomes missed this benchmark (*g* = −0.2; 95% CI: −0.30, −0.09; *p* ≤ .01). Similarly, the aggregated data of the two studies providing alcohol-related outcomes resulted in an almost nil and non-significant effect (*g* = −0.06; 95% CI: −0.2, 0.07; *p* = .36).

When interpreting the above-mentioned results it is important to consider the relationship between mental health and academic performance in general, which is likely complex and warrants inspection over time. This is highlighted in the two-year longitudinal study by Eisenberg et al. (2009), showing that depressive and anxiety symptoms, and even more so their comorbid presentation, are negatively related to GPA and the probability of dropping out of college. The authors mention the importance of chronic depression, which may lead to a vicious circle in which students' depressive symptoms and low judgment of their academic abilities reinforce each other (Eisenberg et al., 2009). In that regard, it is conceivable that improvement in academic performance becomes apparent only after mental health complaints have decreased or have been resolved, or vice versa (Crocker et al., 2003). With this in mind, the small clinical effects of the included e-mental health interventions might at least partly explain their small effect on academic performance. To our knowledge, there is no scientific evidence yet for this from the field of education. However, using occupational therapies for employees with depression as an analogous example, it is not uncommon that such therapies are employed over the course of several months, after which a person can resume work (Hees et al., 2013; Hees et al., 2010).

### 4.2 Strengths and limitations

The results of this meta-analysis need to be interpreted in the context of its strengths and limitations. To begin with, few studies could be included, the primary reason for exclusion being that the majority of RCTs (*N* = 44) did not report on measures of academic performance. One potential explanation is the fact that such measures are not part of the commonly assessed variables in clinical studies, which might be limited to (mental) health-related outcomes. From the students' perspective, being asked to provide or agree to the collection of academic performance data might cast doubt on the institution's intention to test or implement the e-mental health intervention in question. Despite these considerations, the combined sample size was large. Our pre-planned power analysis required the inclusion of around five studies with approximately 160 participants per condition in order to find a small standardized mean difference of *g* = 0.18. Though post-hoc power calculations are discouraged (Hoenig and Heisey, 2001), we can assume that our sample (six studies, on average 200 participants per condition) approached sufficient size to detect the effect of *g* = 0.26. However, we encountered a considerable degree of heterogeneity that we were unable to investigate through subgroup analyses due to the few included studies.

Four of the six included studies targeted incoming or freshmen students with no mentioning of clear clinical in- or exclusion criteria. In the absence of a clear outline of the population, the majority of interventions have to be seen as universal prevention strategies. Taken together with a relatively high baseline level of academic performance, this might have created a ceiling effect that precluded any potential intervention effects. Similarly, the risk of bias of the included studies was high, with the study with the lowest risk of bias (Epton et al., 2014) fulfilling just three out of the five assessed Cochrane risk of bias criteria. In particular, only one study (Epton et al., 2014) evidently concealed group allocation and, as is often the case in non-pharmacological trials (Boutron et al., 2007), none clearly ensured blinding of participants.

A final point of consideration is that unlike questionnaires, which can technically be administered at any given point in time, an assessment of academic performance is often temporally predetermined. That is, independent of when students are asked to provide information on e.g. their exam grade, this information is invariably connected to a fixed point in time, namely the time of the exam or the end of the semester (e.g. for GPA). It can therefore be difficult to plan the temporal distance between the administration of the intervention and the academic performance outcome. As a result, post-intervention assessments of academic performance in the included studies were likely not comparable. Although new concepts, such as presenteeism (i.e. impaired performance due to physical and mental complaints) in students are developed together with associated questionnaires (e.g. Presenteeism Scale for Students, PSS, Matsushita et al., 2011), these are rarely used (e.g. Harrer et al., 2018b). In a similar vein, reports of retention and other long-term indicators of academic success are scarce, potentially because their assessment is sensible only in longitudinal designs (e.g. Arria et al., 2013; Eisenberg et al., 2009).

### 4.3 Implications and future research

In this first meta-analysis, we retained RCTs comparing e-mental health interventions to inactive controls for the pooling of data. As effect sizes are generally larger in these designs (Mohr et al., 2009), subsequent studies are encouraged to compare their interventions to active control groups. In addition, effects may be more pronounced for specific individuals, raising the need for moderator analyses in e.g. individual patient data meta-analyses (Karyotaki et al., 2018).

Furthermore, a set of three important implications emerged, based on the findings summarized above. Firstly, the lack of academic performance measures impeded the inclusion of most studies and should be tackled. Technology offers ways to achieve this with minimal burden on the participant and at the same time warranting the blind assessment of academic performance. Big data approaches are one example for – given the appropriate caution – gathering anonymous data on e.g. GPA at the end of the semester while avoiding potentially biased and burdensome self-reports. We therefore encourage researchers involved in the testing of e-mental health interventions for students to consider routinely including academic performance as an outcome measure. Related to this, implementing more longitudinal studies in order to eliminate the constraints inherent to cross-sectional designs is crucial. The former is important to reliably assess the causal relationship between university students' mental health and their academic performance.

Secondly, attention should be given to the lack of clear criteria that delineate the targeted populations. Researchers are encouraged to use tools such as trial preregistrations and the publication of protocols in order to maintain study quality. Only one study in our meta-analysis did so (Epton et al., 2014; Epton et al., 2013). Checklists, such as the CONSORT-EHEALTH (Eysenbach, 2011) have been developed to aid in that regard. Thirdly, subsequent reviews should expand on the concept of academic performance. Potentially, presenteeism, retention, but even cognitive measures can serve as a proxy for students' ability to perform in college and university.

## 5 Conclusion

To our knowledge, this is the first synthesis of the evidence for the effectiveness of e-mental health interventions on improving academic performance. Only a subset of studies could be included due to a lack of academic performance measures. The pooled effect size was small and non-significant and risk of bias of studies included was high. A comprehensive overview was given and recommendations on including academic performance measures as routine outcomes emerged as a result.

## Registration and PRISMA statement

This meta-analysis has been registered on the public research platform figshare (https://doi.org/10.6084/m9.figshare.7088504.v1). The outline and the search strings that were used can be accessed through www.figshare.com or under digital object identifier (DOI) https://doi.org/10.6084/m9.figshare.7088504. A populated PRISMA checklist (Moher et al., 2001) is provided as supplement to this publication.

## Funding

This study was conceived and executed as part of a PhD trajectory which is funded by the European Commission (10.13039/100010661Horizon 2020 Research and Innovation Action, grant agreement 634757).

## Author contributions

FB, NB, AK, PC, DDE, and HR designed the study. FB and NB performed the searches and data extraction. FB wrote the manuscript. All authors contributed to and approved the manuscript.

## Declaration of competing interest

The authors declare the following financial interests/personal relationships which may be considered as potential competing interests:

Dr. Ebert has served as a consultant to/on the scientific advisory boards of Sanofi, Novartis, Minddistrict, Lantern, Schoen Kliniken, Ideamed and German health insurance companies (BARMER, Techniker Krankenkasse) and a number of federal chambers for psychotherapy. He is also stakeholder of the Institute for health training online (GET.ON), which aims to implement scientific findings related to digital health interventions into routine care.

## Appendix A Supplementary data

PRISMA checklist Image 1

PubMed search string Image 2
