# Supplementary material for: The effect of e-mental health interventions on academic performance in university and college students: A meta-analysis of randomized controlled trials
Source: Internet Interv. 2020 Apr 23;20:100321. doi: 10.1016/j.invent.2020.100321 (PMC7201188; doi:10.1016/j.invent.2020.100321)
Supplement: Supplementary file 2 — PubMed search string [file mmc2.docx]

**Supplementary material**

### *Search string PubMed [Limited to records published between 01-2000 and 09-2019]*

Search (((("Students"[Mesh] OR "Schools, Medical"[Mesh] OR "Universities"[Mesh] OR "Education, Medical, Undergraduate"[Mesh] OR sophomore* [tiab] OR freshm* [tiab] OR student* [tiab] OR universit* [tiab] OR college* [tiab] OR undergraduat* [tiab] OR medical school* [tiab] OR medical education* [tiab] OR (tertiary [tiab] AND (Education*[tiab] OR "Education"[Mesh])) OR tertiary education* [tiab])) AND ("Internet"[Mesh] OR "Mobile applications"[Mesh] OR "Computers"[Mesh] OR "Therapy, Computer-Assisted"[Mesh] OR "Virtual Reality"[Mesh] OR "Virtual Reality Exposure Therapy"[Mesh] OR Internet [tiab] OR mobile application* [tiab] OR Computer* [tiab] OR Computer-assisted [tiab] OR Online [tiab] OR Web-based [tiab] OR E-health [tiab] OR Mobile phone* [tiab] OR Smartphone* [tiab] OR smart phone* [tiab] OR Mobile device* [tiab] OR Tablet* [tiab] OR App* [tiab] OR m-health [tiab] OR ehealth [tiab] OR mhealth [tiab] OR iPad* [tiab] OR iPhone* [tiab] OR blended [tiab] OR icbt [tiab] OR i-cbt [tiab] OR virtual realit* [tiab] OR "virtual reality" [tiab] OR virtualrealit* [tiab] OR VR [tiab] OR "Virtual Reality Exposure Therapy" [tiab])) AND ("Academic Performance"[Mesh] OR "Academic Success"[Mesh] OR "Educational status"[Mesh] OR "Underachievement"[Mesh] OR "Academic Failure"[Mesh] OR academic performance* [tiab] OR academic success* [tiab] OR Educational status [tiab] OR academic achievement* [tiab] OR academic failure* [tiab] OR GPA [tiab] OR grade point average [tiab] OR gradepoint-average [tiab] OR “study related” [tiab] OR “study-related” [tiab] OR educational attainment* OR mark* [tiab] OR grade* [tiab])) AND ("Randomized Controlled Trial" [Publication Type] OR "Randomized Controlled Trials as Topic"[Mesh] OR "Controlled Clinical Trial" [Publication Type] OR randomised trial [tiab] OR randomized trial [tiab])
